# Lower Prevalence of Human Papillomavirus in Head and Neck Squamous Cell Carcinoma in Middle Eastern Population: Clinical Implications for Diagnosis and Prevention

**DOI:** 10.7759/cureus.34912

**Published:** 2023-02-13

**Authors:** Saeeda Almarzooqi, Muhammad Jawad Hashim, Aktham Awwad, Charu Sharma, Dhanya Saraswathiamma, Alia Albawardi

**Affiliations:** 1 Department of Pathology, College of Medicine and Health Sciences, United Arab Emirates University (UAE) University, Al Ain, ARE; 2 Department of Family Medicine, College of Medicine and Health Sciences, United Arab Emirates University (UAE) University, Al Ain, ARE; 3 Department of Laboratory Medicine, Tawam Hospital, Al Ain, ARE; 4 Department of Internal Medicine, College of Medicine and Health Sciences, United Arab Emirates University (UAE) University, Al Ain, ARE

**Keywords:** in situ hybridization, immunohistochemistry, uae, head and neck squamous cell carcinoma, high-risk human papillomavirus

## Abstract

Introduction

Head and neck squamous cell carcinoma (HNSCC) is the sixth most common cancer worldwide. The prevalence of human papillomavirus (HPV) in HNSCC varies across regions.

Objective

This study aimed to determine the prevalence of high-risk HPV (hrHPV) among patients with HNSCC in the Middle East region.

Methods

Samples from patients with oropharyngeal or laryngeal lesions who underwent biopsy or resection at a tertiary care hospital from 2010 to 2015 were collected. Those confirmed as squamous cell carcinoma (SCC) on histopathology were identified as cases (n = 61), whereas benign lesions were used as controls (n = 83). Immunohistochemistry (IHC) for p16, p53, Ki-67, and in situ hybridization (ISH) for hrHPV genotypes 16, 18, 31, 33, 35, 39, 45, 51, 52, 56, 58, and 66 were performed on all cases.

Results

A total of 154 cases were studied: 61 squamous cell cancers (cases), 83 benign lesions (control), and 10 dysplasia specimens. Among the cases, only five (8.6%) were positive for hrHPV, whereas only one control specimen tested positive. The SCC group had higher mean age, male sex, and history of cigarette smoking and alcohol usage. Among the hrHPV-positive SCC cases, 80% had a tumor in the oropharyngeal region. All hrHPV-positive cases were positive for p16 and p53 immunostains.

Conclusion

Among HNSCC cases, hrHPV was detected at a lower rate compared to other regions of the world. This study suggests that hrHPV plays a minor role in the pathogenesis of HNSCC in this region, compared to tobacco use and alcohol consumption.

## Introduction

Head and neck squamous cell carcinoma (HNSCC) is the sixth most common cancer worldwide [1]. Drinking alcohol, chewing tobacco, and smoking have traditionally been considered major risk factors for HNSCC. Other risk factors include ethnicity, genetic background, geographical origin, and nutritional status. Recent studies have shown that human papillomavirus (HPV) infection is one of the emerging determinants for HNSCC, delineating a new subtype of tumor different from HPV negatives [2,3]. HPV is recognized as a carcinogen for HNSCC by the International Agency for Research on Cancer [4]. HPV is epidemiologically classified into three stratification groups: high-risk HPV (hrHPV) group includes 16, 18, 31, 33, 35, 39, 45, 51, 52, 56, 58, and 59; probably hrHPV includes 26, 53, 66, 68, 73, and 82; and lastly, the low-risk group includes 6, 11, 40, 42, 43, 44, 54, 61, 70, 72, 81, and CP6108 [5]. hrHPV turns infected cells into cancerous ones by expressing oncoproteins E6 and E7, which in turn bind to two powerful tumor suppressor genes: p53 and pRB [6]. Globally, the most prevalent subtype is HPV 16, representing 82% of all cases, followed by HPV 18 and then a few other sporadic types [7]. Viral DNA integration into malignant cells is evident via increased expression of the p16 protein. There is a good concordance between cost-effective p16 immunostain expression and positive results of hrHPV using in situ hybridization (ISH) [8].

HPV infection is associated with approximately 30% of HNSCC, and its prevalence is on the rise [6]. Among HNSCCs, oropharyngeal squamous cell carcinomas (SCCs) (OPSCCs) show a high association with HPV infection. The OPSCCs include cancers of the tonsils, soft palate, base of the tongue, and uvula. Patients with OPSCC who are HPV-positive are usually young, sexually active, do not consume alcohol, and do not smoke [9].

HPV infection is also associated with large variations in the health status of human populations [7] that are related to socioeconomic, ethnic, and genetic factors. HPV-associated HNSCC prevalence is different by region because of the differences in social practices endorsed by different cultures [10]. The clinical outcome of HPV-positive tumors is better compared to virus-negative lesions. Patients with HPV-driven HNSCC respond well to radiotherapy and chemotherapy, leading to improved overall survival [11,12].

There are little data available on the prevalence of HPV-related HNSCCs in the Middle East region, particularly in the United Arab Emirates (UAE). Thus, this study aimed to determine the prevalence of hrHPV-driven HNSCC in this region. This study adds information on risk factors for disease prevention and diagnosis.

## Materials and methods

Study design

This case-control study was conducted at a major oncology referral center situated in the city of Al Ain, UAE. Clinical data were obtained from the Tawam Hospital pathology laboratory electronic system. All cases from 2010 to 2015, including both resections and biopsies from the oral cavity, oropharynx, nasopharynx, tonsils, and larynx, were included. A total of 154 samples were identified, and they were divided into three groups: squamous cell carcinoma (SCC) (n = 61), benign lesions (controls, n = 83), and dysplasia (n = 10). The control group included benign lesions and excluded non-SCC malignancies such as lymphoma (n = 3), nasopharyngeal carcinoma (n = 1), rhabdomyosarcoma (n = 1), and undifferentiated carcinoma (n = 1). Patients’ electronic medical records were reviewed to obtain the relevant clinical data such as age, gender, nationality, and history of smoking, tobacco chewing, and alcohol consumption. This study was approved by the Al Ain Medical District Human Research Ethics Committee with approval number 12/92.

Histopathology review

Original histopathology slides were reviewed to confirm the original diagnosis and to ascertain tumor-specific pathological parameters such as histological type, tumor grade, tumor size, and lymph node status. Immunohistochemistry for p16, p53, and Ki-67 and hrHPV in situ hybridization for genotypes 16, 18, 31, 33, 35, 39, 45, 51, 52, 56, 58, and 66 were performed on all 154 cases.

Immunohistochemistry (IHC)

A 4 μm-thick section was cut and mounted on Superfrost Plus slides (Thermo Fischer Scientific, Waltham, MA, USA). Slides were heated for 20 minutes at 70°C. Primary antibodies were replaced by a wash buffer in the negative control slides. p53 and Ki-67 immunostains and endogenous epitopes were unmasked by 20-minute heating of the sections at 97°C in target retrieval solution buffer (pH 9 for p53 and Ki-67) using a pre-treatment link rinse station (Dako, Agilent Technologies, Santa Clara, CA, USA). Then, the immune detection was performed using specific monoclonal antibodies (mouse) against p53 (clone DO-7, 1:1000; Dako, Agilent Technologies, Santa Clara, CA, USA) and Ki-67 (clone MIB-1, 1:400; Dako, Agilent Technologies, Santa Clara, CA, USA). Antibodies were diluted appropriately in antibody diluent (Diamond, Cell Marque, Rocklin, CA, USA). The immunohistochemistry reactions were carried out using Autostainer Link 48 (Agilent Technologies, Santa Clara, CA, USA) automated staining platform and the EnVision FLEX™ visualization system (Agilent Technologies, Santa Clara, CA, USA). Ovarian carcinoma served as a positive control for p53, and tissue from the tonsils served as a positive control for Ki-67. Both Ki-67 and p53 are nuclear markers.

For p16 immunostain, a BenchMark ULTRA autostainer (Ventana Medical Systems, Oro Valley, AZ, USA) was used. Slides were deparaffinized using an EZ prep solution. Antigen retrieval was performed using Cell Conditioning 1 (heat-induced) for 36 minutes at 95°C. Then, the sections were incubated for 16 minutes with Ventana antibody (clone E6H4, CINtec p16 Histology, Roche, Basel, Switzerland) to detect p16. Following incubation with the primary antibody, an amplification kit was applied. UltraView Universal DAB Detection Kit (Ventana Medical Systems, Oro Valley, AZ, USA) was used to detect the specific reactions. Slides were counterstained with hematoxylin. The cellular staining pattern for CINtec p16 is nuclear and/or cytoplasmic.

For HPV detection, chromogenic in situ hybridization (CISH) was performed using a fully automated BenchMark ULTRA staining platform (Ventana Medical Systems, Oro Valley, AZ, USA) with ISH iView Blue Plus Detection Kit and Red Counterstain II (Ventana Medical Systems, Oro Valley, AZ, USA). For this assay, slides were conditioned using Ventana Cell Conditioning 2 (three cycles: mild, 12 minutes; standard, eight minutes; and extended, 12 minutes) and ISH protease 3 (four minutes). Denaturation for 12 minutes was followed by hybridization for two hours using the HPV III Family 16 probe (B) set, which captures HPV genotypes 16, 18, 31, 33, 35, 39, 45, 51, 52, 56, 58, and 66.

ISH iView Blue Plus Detection Kit was used for signal detection, which is an indirect biotin-streptavidin system to detect fluorescein-labelled probes. The kit uses an alkaline phosphatase enzyme and nitro blue tetrazolium (NBT)/5-bromo-4-chloro-3-indolyl phosphate (BCIP) substrate chromogen reaction and provides an intense blue, a permanent color, and a red counterstain. All reagents were pre-diluted and ready to use on BenchMark Series automated slide stainers. The presence of hrHPV was scored positive when either a large homogenous navy blue precipitate was present (episomal pattern) or discrete stippled navy blue dots (integrated pattern) were identified within the nuclei of malignant cells. Suitable positive controls of high-grade cervical intraepithelial neoplasia (CIN) and negative controls were also included.

Statistical analysis

Data analysis was conducted using the current version (version 28) of Statistical Package for Social Sciences (SPSS) (IBM SPSS Statistics, Armonk, NY, USA). Statistical tests included the chi-square test, Pearson correlation coefficient, and multivariate regression analysis. An alpha level of less than 0.05 was considered statistically significant. Logistic regression analysis was used to evaluate clinical predictors of malignant lesions compared to controls (10 cases with dysplasia were excluded in this analysis; 130 cases were included after excluding those with missing values).

## Results

A total of 154 samples were studied, which included SCC (cases, n = 61), benign lesions (controls, n = 83), and dysplasia (n = 10). Among them, 42.9% were UAE nationals, and the rest were from other Arab countries and the South Asian region. Table 1 summarizes the study population demographics and risk factors. The mean (standard deviation, SD) age in the SCC group was 59.4 (13.1) years (range: 27-84 years); in the control group, it was lower at 38.4 (17.5) years and in patients with dysplasia 58.4 (11.2) years (p < 0.001). The majority of patients with SCC (75.4%) were males, compared with 48.2% of patients in the control group and 40% of patients with dysplasia. In the SCC group, UAE nationals represented 30.5%, and the rest were expatriates. These included patients from Arab countries such as Egypt, Syria, Lebanon, Morocco, Sudan, and Palestine (28.8%) and patients from South Asian countries such as India, Pakistan, and Bangladesh (30.5%).

**Table 1:** Demographic characteristics of patients with HNSCC “n” indicates the number of cases, and “%” indicates the percentage of total cases. Values are based on two-tailed chi-square tests (except for age, which was analyzed using a one-way ANOVA test) SCC, squamous cell carcinoma; HNSCC, Head and neck squamous cell carcinoma; SD, standard deviation; UAE, United Arab Emirates; ANOVA, analysis of variance

Characteristics	SCC % (n = 61)	Dysplasia % (n = 10)	Control (benign lesions) % (n = 83)	Total % (n = 154)	P-value
Gender					0.002
Female	24.6 (15)	60.0 (6)	51.8 (43)	41.6 (64)	
Male	75.4 (46)	40.0 (4)	48.2 (40)	58.4 (90)	
Age in year (mean ± SD)	59.4 (13.1)	58.4 (11.2)	38.4 (17.5)	-	<0.001
Nationality					0.011
UAE	30.5 (18)	20(2)	55.4 (46)	42.9 (66)	
Other Arabs	28.8 (17)	20(2)	13.3 (11)	21.4 (33)	
South Asian	30.5 (18)	40(4)	15.7 (13)	20.8 (32)	
Others	10.2 (6)	20(2)	15.7 (13)	13.6 (21)	
Tobacco smoking	38.3 (23)	0.0 (0)	13.4 (11)	-	<0.001
Tobacco chewing	5.0 (3)	10.0 (1)	1.2 (1)	-	0.215
Alcohol intake	16.7 (10)	0.0 (0)	2.4 (2)	-	0.005
Surgical procedure					0.005
Biopsy	70.5 (43)	100.0 (10)	89.2 (74)	-	
Resection	29.5 (18)	0.0 (0)	10.8 (9)	-	
Anatomic location					<0.001
Larynx	29.5 (18)	20.0 (2)	4.8 (4)	-	
Oral cavity	62.3 (38)	70.0 (7)	66.3 (55)	-	
Pharynx	8.2 (5)	10.0 (1)	28.9 (24)	-	

Tumor characteristics

**Figure 1:**
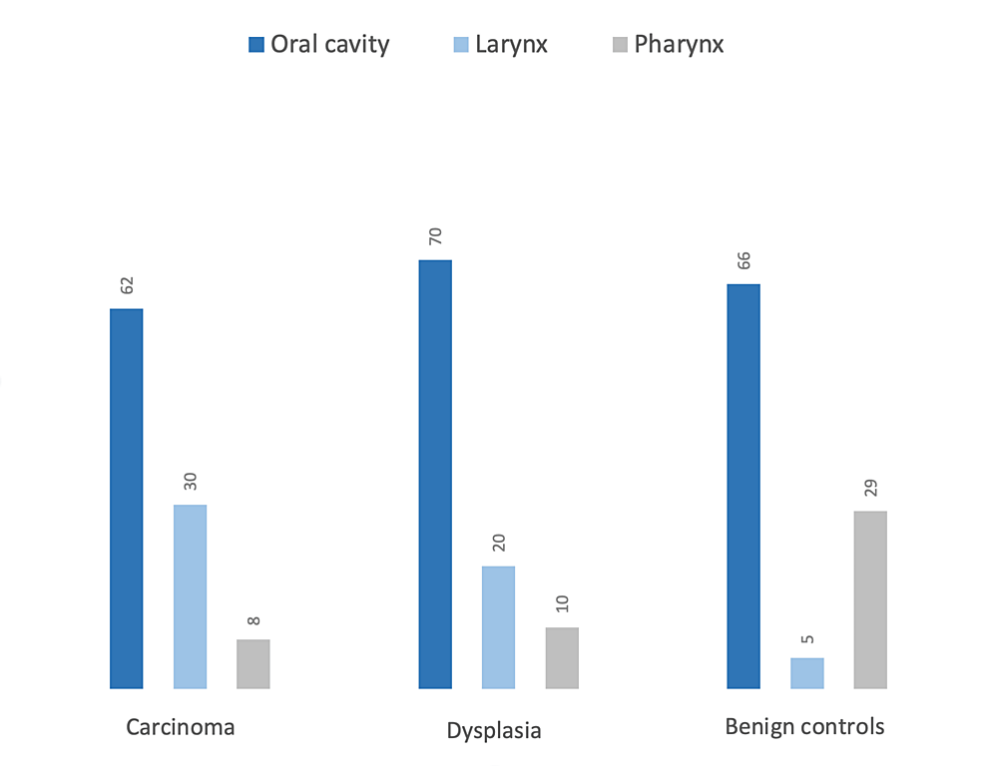
Anatomical distribution of head and neck lesions Numbers indicate percentages within each group

The proportion of specimens positive for SCC was 61/154 (40%) in our study. The distribution of the primary location of the tumor was as follows: the larynx (29.5%), the oral cavity including the buccal mucosa, the floor of the mouth and gingiva (52.5%), and the pharynx (8.2%). Tumor size estimate was available in 32 from 61 SCC cases in which 70.5% of samples in the SCC group were collected through biopsy and the rest by resection. Most SCC cases were moderately differentiated (51.7%); well-differentiated SCC formed 40.0%, whereas poorly differentiated formed 8.3% (Figure 1).

SCC and hrHPV

Table 2 outlines the characteristics of SCC tumors with respect to hrHPV status. Among the 53 SCC cases and 69 controls, five cases proved positive for hrHPV by in situ hybridization, which represents 8.6% of the SCC cases. One control case also tested positive for hrHPV, which represented 1.4% of the total control. The hrHPV test was not feasible on some specimens due to insufficient tissue in the paraffin blocks. The mean age of the hrHPV-positive patients with SCC was slightly higher but not statistically significant at 62.2 years (SD: 14.8), with all cases over 50 years (p = 0.12). One hrHPV-positive case was in the tongue, and the remaining four arose from the tonsils. By comparison, none of the samples in the control group were positive for hrHPV.

**Table 2:** Clinical characteristics of patients with and without hrHPV ISH 16/18 “n” indicates the number of cases, and “%” indicates the percentage of total cases hrHPV, high-risk human papillomavirus; ISH, in situ hybridization; UAE, United Arab Emirates

Characteristics	hrHPV ISH 16/18 status		
	Positive % (n)	Negative % (n)	P-value
Histological grade			0.127
Well-differentiated	0.0(0)	43.4 (23)	
Moderately differentiated	75.0 (3)	50.9 (27)	
Poorly differentiated	25.0 (1)	5.7 (3)	
Tumor location			0.009
Larynx	0.0 (0)	16.9 (23)	
Oral cavity	33.3 (2)	66.2 (90)	
Pharynx	66.7 (4)	16.9 (23)	
National origin			0.892
UAE	33.3 (2)	43 (58)	
South Asian	33.3 (2)	21.5 (29)	
Non-UAE Arab	16.7 (1)	22.2 (30)	
Others	16.7 (1)	13.3 (18)	
Gender			
Female	33.3 (2)	41.2 (56)	0.702
Male	66.7 (4)	58.8 (80)	
Smoking	50 (3)	22.4 (30)	0.119
Tobacco chewing	0 (0)	3.7 (5)	0.630
Alcohol	16.7 (1)	8.2 (11)	0.469

Immunohistochemistry for p16, p53, and Ki-67

**Figure 2:**
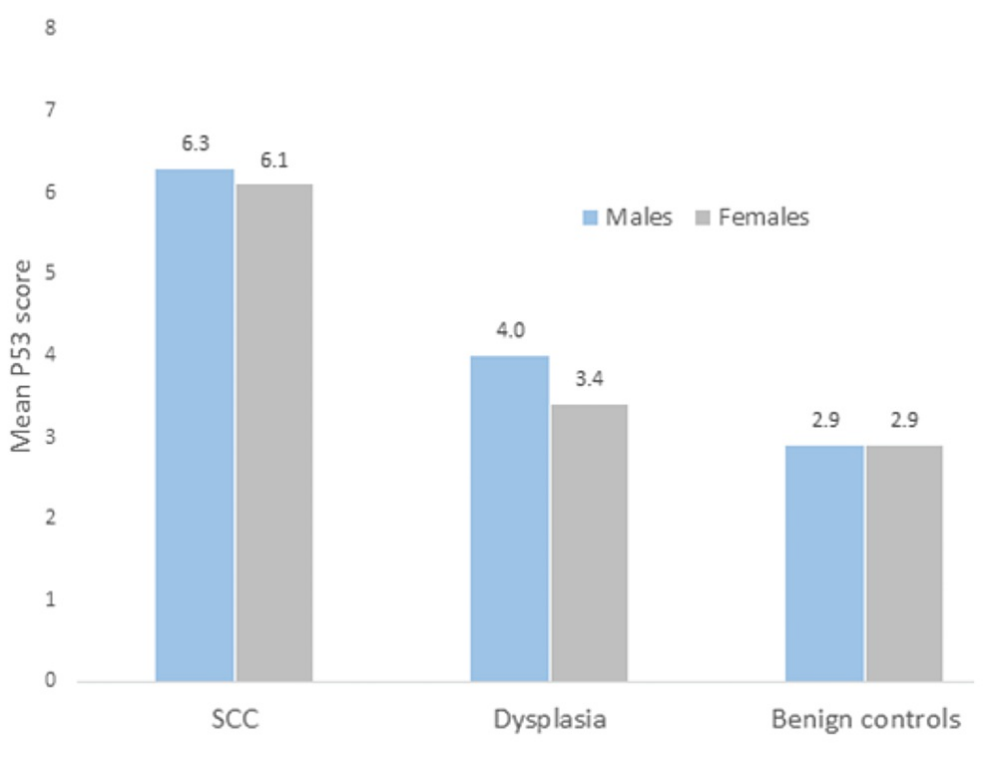
p53 scores in SCC, dysplasia, and benign lesions by sex Numbers indicate mean p53 scores in each group SCC: squamous cell carcinoma

All hrHPV-positive cases were also positive for p16. Moreover, hrHPV-positive cases were also stained with p53 immunostain. All cases positive for hrHPV were also positive for p53. p53 scores in SCC cases were in the higher range compared with the other two groups (Figure 2). The mean Ki-67 expression in the SCC cohort of cases was 55%, indicating that the Ki-67 in noncancerous tissue does not have clinical significance. hrHPV-positive SCC cases displayed a slightly higher proliferation rate with Ki-67 labelling. Among the hrHPV-positive cases, the mean for Ki-67 index labelling was 74% (range: 50%-90%). However, there is no significant correlation between the Ki-67 index and hrHPV positivity (p = 0.7).

On multivariate analysis, the only statistically significant predictor of hrHPV positivity was p16 immunostaining, whereas other variables such as age, gender, smoking, chewing tobacco, alcohol use, tumor grade, location, and p53 staining were not significant (stepwise binary logistic regression, Cox R^2^ = 0.27).

Risk factor assessment

The prevalence of tobacco smoking was 38.3% in the SCC group, whereas 5% had a history of tobacco chewing, and 16.7% reported a history of alcohol intake; in comparison, the percentages for the control group were lower at 13.4%, 1.2%, and 2.4%. There was a statistically significant association between the history of smoking and alcohol intake with the development of SCC (p = 0.003). However, there was no statistically significant association between SCC and tobacco chewing possibly due to the small numbers (n = 4) in this subgroup. On multivariate analysis, statistically significant predictors included age (adjusted odds ratio: 1.085) and location of the lesion but not gender, cigarette smoking, chewing tobacco, or consuming alcohol (Nagelkerke R^2^: 0.60).

## Discussion

Human papillomavirus prevalence in HNSCC is increasing worldwide and varies significantly across countries and regions [13,14]. During the past decade, HPV-induced HNSCCs has increased by 36.5% globally, and HPV-induced OPSCCs constitute 71% and 51.8% of all OPSCCs in the USA and the United Kingdom, respectively [2,15]. As hrHPV involvement in HNSCC varies widely geographically, this study provides relevant information on HNSCC in the Middle East region. This is the first study in the UAE to examine the link between hrHPV and HNSCC and other risk factors. It provides data on HPV-positive genotypes 16, 18, 31, 33, 35, 39, 45, 51, 52, 56, 58, and 66 using in situ hybridization.

In this study, five SCCs tested positive for hrHPV, representing 8.6% of all SCCs. It remains below the worldwide prevalence rate of 30%-40%. The prevalence rate of HPV-induced HNSCCs is 3.5% in Saudi Arabia, confirming the geographical variability in the development of these tumors [7]. HPV-induced HNSCC appears to be of less importance in Eastern Asia (2%) and Europe (6%) than in the well-developed Western countries (30%-40%), where most studies are reported [16,17].

In this study, the highest occurrence of HPV was observed in oropharyngeal cancers (80%), primarily in the tonsils (4/5). In 20% (1/5) of HPV-positive cases, the tumor was in the tongue, whereas none of the other oral or laryngeal SCCs were hrHPV-positive. These results are in line with other published reports that HPV-induced OPSCCs are now accounting for 70%-90% of total OPSCCs in many parts of the world [10,18,19]. The papillomavirus-associated HNSCCs typically occur in the oropharynx’s palatine and lingual tonsils, whereas tobacco-associated HNSCCs generally occur in the mouth, larynx, and hypopharynx [1].

Smoking and alcohol consumption are considered influential risk factors for HNSCC. Over the past few years, smoking prevalence has declined in most high-income countries, but HPV infection has emerged as an important risk factor that has increased the incidence of HNSCC [19,20]. In this study, a history of tobacco smoking and alcohol intake was significantly correlated with the development of HNSCC. This association is well-established, and the duration of smoking and consumption rate are also important [15]. Conversely, tobacco chewing showed no statistical significance when correlated with HNSCC (p = 0.38), probably because of the small number of such cases among the samples.

According to this study, all hrHPV-positive SCC cases occurred in patients over the age of 50 years (range: 51-84 years). These data contrast with most reports of HPV-associated SCCs, which tend to occur in a younger age group who are sexually active males, consume little or no alcohol, and are nonsmokers [9,21]. Long-term tobacco use and elderly age could be the contributing risk factors [22].

HPV plays a role as an etiologic agent in cervical cancer. It is transmittable sexually, and HPV-positive HNSCC is also associated with sexual behavior, oral sex, and multiple sex partners [23-25]. Because of the lack of longitudinal data, no confident statements can be made regarding changes in sexual practices or an increase in the incidence of hrHPV-associated HNSCC in the study population. Our findings demonstrate that HPV-driven HNSCC has a very low prevalence in our population. The main reason for the low rate could be attributed to the difference in sociocultural lifestyles and sexual practices [26-28]. In spite of the UAE’s mixed nationality, most of its citizens belong to South Asia (54%) and other Arab countries (12%), with similar sociocultural values.

It is important to mention that cervical cancer caused by HPV is one of the top three malignancies affecting Emirati females [29]. The reported incidence of abnormalities in cervical screening in the UAE was 3.6% [30]. Consequently, in the year 2008, the UAE government launched a national HPV vaccination program, targeting school females and females aged 15-26 years [31]. Both the bivalent (HPV 16 and HPV 18) and subsequently the quadrivalent vaccines (HPV 6, HPV 11, HPV 16, and HPV 18) were used. In this region, there will be little, if any, prospective benefit from HPV vaccines in reducing the incidence of HNSCC. A more effective approach would be to implement tobacco smoking awareness campaigns, a total ban on tobacco use in public places, higher taxation on tobacco, and strict regulations for cafes and restaurants that serve shisha (tobacco water pipe) to customers.

Limitations

This was a retrospective study conducted in a single institute with a relatively small sample size. The exact representation of HPV-induced HNSCC may vary across different emirates in the UAE. The study can be further extended to other oncology centers across different emirates in the UAE with more molecular testing including epidermal growth factor receptors.

## Conclusions

The prevalence of hrHPV was lower (8.6%) in our study population compared to the reported global HPV prevalence (30%-40%). This finding differs from the epidemiology of HNSCC in many other geographical regions, especially in the Western population. Both smoking and alcohol appear as risk factors for oral cancers in this study population. This has direct implications for the prevention of head and neck cancers in the region. This study provides data from the UAE population, and the findings of this study would be useful to healthcare providers, leaders, and policymakers.

## Appendices

Abbreviations

CIN, cervical intraepithelial neoplasia; CISH, chromogenic in situ hybridization; HNSCC, head and neck squamous cell carcinoma; HPV, human papillomavirus; hrHPV, high-risk human papillomavirus; IARC, International Agency for Research on Cancer; IHC, immunohistochemistry; ISH, in situ hybridization; OPSCC, oropharyngeal squamous cell carcinoma; PCR, polymerase chain reaction; SCC, squamous cell carcinoma; SD, standard deviation; TNM, tumor-node-metastasis
